# 42 Utilizing FAB Scores to Determine Next Level of Care for Patients with Burn Injury

**DOI:** 10.1093/jbcr/irad045.016

**Published:** 2023-05-15

**Authors:** Teresa Geib, David Hill, Meghan Mincey, Sai Velamuri, Sandra Fletchall

**Affiliations:** Firefighters Burn Center, Memphis, Tennessee; Regional One Health, Memphis, Tennessee; Firefighters Burn Center, Memphis, Tennessee; University of Tennessee, Memphis, Tennessee; Firefighters Burn Center, Memphis, Tennessee; Firefighters Burn Center, Memphis, Tennessee; Regional One Health, Memphis, Tennessee; Firefighters Burn Center, Memphis, Tennessee; University of Tennessee, Memphis, Tennessee; Firefighters Burn Center, Memphis, Tennessee; Firefighters Burn Center, Memphis, Tennessee; Regional One Health, Memphis, Tennessee; Firefighters Burn Center, Memphis, Tennessee; University of Tennessee, Memphis, Tennessee; Firefighters Burn Center, Memphis, Tennessee; Firefighters Burn Center, Memphis, Tennessee; Regional One Health, Memphis, Tennessee; Firefighters Burn Center, Memphis, Tennessee; University of Tennessee, Memphis, Tennessee; Firefighters Burn Center, Memphis, Tennessee; Firefighters Burn Center, Memphis, Tennessee; Regional One Health, Memphis, Tennessee; Firefighters Burn Center, Memphis, Tennessee; University of Tennessee, Memphis, Tennessee; Firefighters Burn Center, Memphis, Tennessee

## Abstract

**Introduction:**

Determining next level of care for burn patients is a difficult task for the entire medical team. Secondary to the nature of the injury, patients often do not have the social or economic support required to return to their prior level of function. The Functional Assessment for Burns (FAB) score has been shown an objective measure to determine functional independence and a predictor of discharge outcomes in adults. However, it is undetermined whether this assessment can be utilized to guide next level of care and time which it takes to return to prior level of function. We hypothesized the number of therapy sessions to be positively associated with patients’ return to baseline FAB.

**Methods:**

This single center, retrospective study of patients admitted to a single ABA-verified burn center was completed by collecting FAB assessment scores within 48 hours of admission and within 48 hours of discharge over the last year. Demographics, injury characteristics, surgical procedures, and number of treatment sessions were collected. Shapiro-wilk test was utilized to test for normality. Regression analysis was utilized to analyze variable associations with changes in FAB scores. A paired t test was used to compare paired FAB scores between admission and discharge.

**Results:**

One-hundred patients were included with a median age of 53 (38.5, 64) years and 7 (3.5, 14) percent total body surface area burned (TBSA). The median FAB for preadmission (preadm.), admission (adm.), and discharge (DC) were 35 (35, 35), 17 (14, 24), and 24 (19, 32), respectively. The paired difference between adm. and DC scores (< 0.001) indicated significant improvement. TBSA and age, together, were significant predictors of change in preadm. to adm. FAB (r2=0.202, p< 0.001). Adm. FAB and TBSA were both associated with number of therapy sessions. Only 16% of patients returned to preadm. level of functional independence by discharge with only 20% qualifying for / entering a post-acute inpatient rehabilitation facility. Number of combined therapy sessions was not associated with return to preadm. FAB. Of note, only 28% returned within 90% of preadmission FAB by discharge. In fact, 13% actually saw their FAB regress from adm. to DC. None of the collected variables were associated with the negative change in FAB scores through admission.

**Conclusions:**

In this sample, number of sessions was not predictive of return to preadmission FAB; however, this may be due to the low numbers that achieved the milestone at all. Despite this fact, there was an alarmingly small number admitted to a subsequent inpatient rehabilitation facility. There was a significant improvement in paired scores from admission to discharge. Taken together, there may be value in reassessing inpatient rehabilitation facility adm. criteria.

**Applicability of Research to Practice:**

FAB scores may be a useful indicator of further rehabilitation needs.

